# Erythritol as a single carbon source improves cultural isolation of *Burkholderia pseudomallei* from rice paddy soils

**DOI:** 10.1371/journal.pntd.0007821

**Published:** 2019-10-21

**Authors:** Trung T. Trinh, Karoline Assig, Quyen T. L. Tran, André Goehler, Linh N. H. Bui, Claudia Wiede, Bettina Folli, Sabine Lichtenegger, Tinh T. Nguyen, Gabriel E. Wagner, Christian Kohler, Ivo Steinmetz

**Affiliations:** 1 Institute of Microbiology and Biotechnology, Vietnam National University, Hanoi, Vietnam; 2 Institute of Hygiene, Microbiology and Environmental Medicine, Medical University Graz, Graz, Austria; 3 Friedrich Loeffler Institute of Medical Microbiology, University Medicine Greifswald, Greifswald, Germany; National University of Singapore, SINGAPORE

## Abstract

**Background:**

Isolation of the soil bacterium *Burkholderia pseudomallei* from tropical environments is important to generate a global risk map for man and animals to acquire the infectious disease melioidosis. There is increasing evidence, that the currently recommended soil culture protocol using threonine-basal salt solution with colistin (TBSS-C50) for enrichment of *B*. *pseudomallei* and Ashdown agar for subsequent subculture lacks sensitivity. We therefore investigated, if the otherwise rarely encountered erythritol catabolism of *B*. *pseudomallei* might be exploited to improve isolation of this bacterium from soil.

**Methodology/Principal findings:**

Based on TBSS-C50, we designed a new colistin-containing medium with erythritol as the single carbon source (EM). This medium was validated in various culture protocols by analyzing 80 soil samples from 16 different rice fields in Vietnam. *B*. *pseudomallei* enrichment was determined in all culture supernatants by a specific quantitative PCR (qPCR) targeting the type three secretion system 1. 51 out of 80 (63.8%) soil samples gave a positive qPCR signal in at least one of the culture conditions. We observed a significantly higher enrichment shown by lower median cycle threshold values for *B*. *pseudomallei* in a two-step culture with TBSS-C50 for 48 h followed by EM for 96h compared to single cultures in TBSS-C50 for either 48h or 144h (p<0.0001, respectively). Accordingly, *B*. *pseudomallei* could be isolated on Ashdown agar in 58.8% (30/51) of samples after subcultures from our novel two-step enrichment culture compared to only 9.8% (5/51) after standard enrichment with TBSS-C50 for 48h (p<0.0001) or 25.5% (13/51; p<0.01) after TBSS-C50 for 144h.

**Conclusions/significance:**

In the present study, we show that specific exploitation of *B*. *pseudomallei* metabolic capabilities in enrichment protocols leads to a significantly improved isolation rate of this pathogen from soil compared to established standard procedures. Our new culture method might help to facilitate the creation of environmental risk maps for melioidosis in the future.

## Author summary

Melioidosis is a vastly underdiagnosed infectious disease caused by the bacterial soil pathogen *B*. *pseudomallei*. Global environmental suitability for *B*. *pseudomallei* has recently been predicted to cover huge areas with three billion people potentially at risk to acquire infection. Although sensitive as well as specific molecular methods are available, cultural isolation of *B*. *pseudomallei* strains from the environment will be indispensable to verify this prediction in potential endemic, low-income countries and is a prerequisite to assess the virulence potential and genetic diversity of strains. The currently applied cultural detection lacks sensitivity, which is at least partly due to a lack in selectivity allowing other highly abundant soil microorganisms to outcompete *B*. *pseudomallei*. We therefore aimed to develop a more selective medium by taking advantage of *B*. *pseudomallei* erythritol catabolism, a metabolic pathway, which is not commonly encountered in other related bacteria. Validation of our new medium with erythritol as the single carbon source was performed by molecular and cultural detection using soil samples from rice paddies in Vietnam. We observed an enhanced enrichment and selectivity resulting in the isolation of *B*. *pseudomallei* strains from soil samples, which were otherwise culture-negative when tested by the standard method.

## Introduction

*B*. *pseudomallei* is a saprophytic soil bacterium causing melioidosis with an estimated global disease burden of 165.000 cases per year, from which 89.000 die [1]. Through the analysis of the potential environmental suitability for *B*. *pseudomallei* it was predicted, that this pathogen exists in many parts of the world where neither the disease melioidosis has been reported nor the respective pathogen has been isolated from the environment. There is a need to provide evidence for the potential occurrence of this pathogen in those regions, but also to more precisely define origins of infections in known endemic areas, since it is known that the pathogen is not evenly distributed in the environment [2]. We have recently shown that direct culture-independent testing of soil samples by using *B*. *pseudomallei*-specific multiple target qPCRs is an useful for environmental *B*. *pseudomallei* screenings and significantly more sensitive compared to current culture methods [3]. Moreover, we could demonstrate a correlation of high quantity of *B*. *pseudomallei*-specific DNA in soil with *B*. *pseudomallei* cultural isolation using the currently recommended culture protocol [3, 4]. This recommended consensus method for soil cultures depends on an enrichment step in threonine-basal salt solution with colistin (TBSS-C50) and subsequent subculture on Ashdown agar to isolate *B*. *pseudomallei* with typical growth characteristics. Although those media contain antibiotics to enhance selectivity for *B*. *pseudomallei*, many other environmental bacteria share the natural resistance against aminoglycosides and polymyxins and therefore can outcompete *B*. *pseudomallei* in the liquid enrichment step as well as on Ashdown agar [5, 6]. Therefore, the higher sensitivity of direct molecular testing is not surprising and also supported by recent studies from Laos, showing that PCR-testing of supernatants from TBSS-C50 enrichment cultures revealed a much higher presence of *B*. *pseudomallei* DNA compared to the subsequent isolation rate of strains on agar [7]. This obviously high rate of false negative cultures needs to be addressed, since the direct detection of *B*. *pseudomallei* DNA in soil samples [3] or in an enrichment culture [7, 8] is an important first step to identify risk areas, but cannot substitute for the isolation of strains for virulence analyses, determination of resistance and molecular epidemiological studies. Furthermore, simple but more sensitive environmental cultures are more likely to be applied in many potential endemic and resource-limited parts of the world, than costly molecular methods.

Previous work on the metabolic capabilities of members of the genus *Burkholderia* showed that *B*. *pseudomallei* can assimilate erythritol whereas other related species such as *B*. *thailandensis* and other *Burkholderia* spp. cannot metabolize this sugar alcohol [9]. Since erythritol catabolism does not seem to be widespread among *Burkholderia* spp. [9] we aimed to developed a new colistin-containing culture medium for the isolation of *B*. *pseudomallei* from soil samples with erythritol as the single carbon source. The selectivity of the medium was assessed by growth experiments with a number of bacteria of the genus *Burkholderia*. We then validated the new medium in various culture protocols in comparison to the currently recommended procedure through attempts to isolate *B*. *pseudomallei* from rice paddy soils collected in the central part of Vietnam. Apart from very early environmental work in Vietnam, providing evidence for the saprophytic nature of *B*. *pseudomallei* [10], there are only few more recent studies on the environmental presence of *B*. *pseudomallei* in northern and southern Vietnam [3, 11–13]. There were no previously published reports on environmental presence of *B*. *pseudomallei* in the central part of Vietnam where the soil samples of this study were collected.

## Materials and methods

### Ethics statement

Permission for soil sampling was obtained from the Ministry of Science and Technology of Vietnam in the context of grant number NVQG2018-08 and oral informed permission was obtained from landowners.

### Bacterial strains

The *B*. *pseudomallei* strains and other bacterial isolates used for growth experiments are listed in Table 1.

**Table 1:** Bacterial strains.

Strain	Species	Source and Origin
K9624	*B*. *pseudomallei*	Human, Thailand
E8	*B*. *pseudomallei*	Soil, Thailand
E212	*B*. *pseudomallei*	Soil, Thailand
770429	*B*. *pseudomallei*	Soil Niger
NCTC4846	*B*. *pseudomallei*	Monkey, Singapore
NCTC8016	*B*. *pseudomallei*	Sheep, Australia
PITT521	*B*. *pseudomallei*	Human, Pakistan
MK441	*B*. *pseudomallei*	Monkey, Philippines
M1831	*B*. *pseudomallei*	Monkey, Indonesia
NCTC10276	*B*. *pseudomallei*	Human, Bangladesh
NCTC1688	*B*. *pseudomallei*	Rat, Malaysia
NCTC7383	*B*. *pseudomallei*	Human, Burma
E264	*B*. *thailandensis*	Soil, Thailand
E27	*B*. *thailandensis*	Soil, Thailand
E201	*B*. *thailandensis*	Soil, Thailand
E232	*B*. *thailandensis*	Soil, Thailand
R-15274	*B*. *vietnamiensis*	Human, Germany
LMG7000	*B*. *stabilis*	Human, Sweden
LMG14291	*B*. *stabilis*	Human, Belgium
R-15280	*B*. *multivorans*	Human, Germany
R-15281	*B*. *multivorans*	Human, Germany
LMG6889	*B*. *cepacia*	
187550	*B*. *cepacia*	Human, Germany
K56-2	*B*. *cencocepacia*	Human, Canada
H111 WT7100	*B*. *cencocepacia*	Human, Germany

### Preparation of culture media

Threonine-basal salt solution [14] containing colistin (TBSS-C50) was prepared according to Limmathurotsakul and colleagues [4]. The TBSS-C50 base consists of 0.45g KH_2_PO_4_, 1.73g K_2_HPO_4_, 0.12g MgSO_4_ * 7H_2_O, 0.02g CaCl_2_ *2H_2_O, 10g NaCl, 0.2g Nitrilotriacetic as well as 20ml of salt solution (see below) solved in 900ml distilled water. After adjusting the pH to 7.2 using 1N KOH, the TBSS-C50 base was sterilized by autoclaving at 121°C for 20min. In a next step, sterile filtered L-threonine solution containing 5.96g L-threonine (BioBasic, Canada) in 100ml distilled water was added to the base. Finally, one million International Units powder of Colistimethate sodium (Colomycin, Forest Laboratories UK Limited, UK) was dissolved in 2ml autoclaved medium and added to the enrichment broth just before usage leading to approximately 50 mg/l of colistin according to Limmathurotsakul and colleagues [4]. To prepare the salt solution 2.31ml H_3_PO_4_ 85%, 0.56g FeSO_4_*7H_2_O, 0.30g ZnSO_4_*7H_2_O, 0.02g CuSO_4_*5H_2_O, 0.13g MnSO_4_*H_2_O, 0.03g Co(NO_3_)_2_*6H_2_O, 0.03g Na_2_MoO_4_*2H_2_O, 0.06g H_3_BO_3_ were added to 1000ml distilled water, solved by constant stirring as well as heating and finally autoclaved at 121°C for 20min. All of the salts and reagents were purchased from either Sigma or Merck suppliers.

To obtain erythritol medium (EM), TBSS-C50 was modified by replacing L-threonine and nitrilotriacetic acid with erythritol and NH_4_H_2_PO_4_. The EM base consists of 0.45g KH_2_PO_4_, 1.73g K_2_HPO_4_, 0.12g MgSO_4_ * 7H_2_O, 0.02g CaCl_2_ *2H_2_O, 10g NaCl and 2.15g NH_4_H_2_PO_4_ supplemented with 20ml of the same salt solution as above, filled up to a final volume of 900ml distilled water and adjusted to a pH of 7.2. After autoclaving and cooling down, the EM base was supplemented with 4g of sterile filtered pre-solved erythritol (BioBasic, Canada) in 100ml deionized water and finally Colistimethate sodium was added as described above.

For first step soil cultures, TBSS-C50 and EM were produced at double strength concentration increasing the concentration of all compounds two fold. For bacterial growth experiments TBSS-C50 and erythritol medium (EM) contained 50mg/l of colistin sulfate (Carl Roth, Germany) instead of Colistimethate sodium. Ashdown agar was prepared as described [15] containing 10g trypticase soy agar (Becton Dickinson, USA), 40ml glycerol (Fisher Chemical, UK), 5mg crystal violet (Sigma-Aldrich, India), 50mg neutral red (Sigma-Aldrich, USA) supplemented with 5mg gentamicin (Carl Roth, Germany) per liter.

### Bacterial growth experiments

Bacterial strains were grown under aerobic conditions on Columbia agar containing 5% sheep blood (BD Biosciences, Austria) at 37°C for 24h and 48h for slow growing cultures, respectively. For subsequent growth kinetics, bacteria were inoculated in either TBSS-C50 broth containing colistin 50mg/l or in erythritol medium with colistin 50mg/l (EM) or without the antibiotic. Turbidity measurements were initiated by inoculation of 200μl medium to an OD_580_ start value of approximately 0.05 and performed in a Bioscreen C (Labsystems, Helsinki, Finland) with hourly measurements at 580nm. Microtitre plates were incubated for 144h at 40°C under continuous shaking.

### Soil samples

During the rainy season in 2018, two soil sampling trips were conducted in the central part of Vietnam. In June, the first sampling included nine sites in north central provinces of Ha Tinh (six sites) and Thanh Hoa (three sites), where many cases with melioidosis have been recently reported [16]. The second sampling in July involved seven sites in south central provinces of Quang Ngai (two sites), Binh Dinh (three sites), Phu Yen (one site) and Khanh Hoa (one site), where no melioidosis cases have been reported so far. All of the sampling sites were rice fields, selected based on previous nationwide *B*. *pseudomallei* environmental surveillance studies performed in 2015 and 2016. The distance between two consecutive sites was in range of 12 to 73km (average of 32km). At each sampling site, five soil samples 10 to 20m apart were collected at a depth of 30cm using a steel auger. Between samplings, the auger was cleaned with bottled water and disinfected with 70% alcohol. Approximately 200g of soils were collected and put in a plastic zip bag using single use cutlery. Relative contents of clay, silt and sand were determined from a homogenized soil sample of each of the 16 sites by sedimentation after removing organic matter with 30% H_2_O_2_ treatment. Soil textures were classified by referencing the textural triangle [17]. Soil textures varied from sandy loam (n = 8), sandy clay loam (n = 3), clay (n = 2) to loam (n = 2) and clay loam (n = 1). Soil samples were stored at ambient temperature and transferred immediately into the laboratory at the Institute of Microbiology and Biotechnology, Vietnam National University, Hanoi.

### Soil cultures and bacterial counts

Soil samples were homogenized before comparative analyses of different culture approaches. Therefore, 20g of soils were weighed into a 100ml Erlenmeyer flask and 20ml of sterilized tap water was added prior to orbital shaking at 160rpm for 1h. The homogenized soil suspension was split into two 50ml tubes. Each soil suspension was filled up with 10ml of double strength concentration of either TBSS-C50 or EM.

The tubes were incubated statically at 40°C with loosely closed caps. Following 48h of incubation, the suspensions were mixed vigorously for 10sec and left for 1h for sedimentation. For subsequent culture, 1ml of either TBSS-C50 or EM cultures was transferred to new tubes containing either 9ml of TBSS-C50 or 9ml of EM medium and incubated for 96h at 40°C. Additionally, the original 48h cultures of TBSS-C50 and EM were kept and further incubated. This resulted in a set of eight bacterial culture conditions for each soil sample. The bacterial cultures included 48h TBSS-C50 and EM cultures, 48h TBSS-C50 plus either 96h TBSS-C50 or 96h EM cultures, 48h EM plus either 96h TBSS-C50 or 96h EM, 144h TBSS-C50 and EM cultures. Culture tubes were mixed vigorously before subsequent molecular analysis and subcultures on Ashdown agar after 48h and 144h of incubation. Aliquots of 0.5ml culture supernatants of each enrichment for qPCR analyses (see below) were centrifuged at 7.000g for 10min. Pellets were stored at -20°C. For subcultures on Ashdown agar, 0.4ml of each culture supernatant was mixed with 0.1ml glycerol to obtain a final glycerol concentration of 20% and frozen at -70°C. All tubes for DNA or Ashdown agar analyses were pseudonymized by giving a unique number to each tube. Bacterial suspensions were thawed and tenfold serial dilutions were plated on Ashdown agar and incubated at 40°C for four days. *B*. *pseudomalle*i colonies were identified based on their typical growth characteristics on Ashdown agar [6, 18, 19]. In contrast to direct isolation of *B*. *pseudomallei* from clinical specimens where different colony morphologies can be observed, the morphotypes obtained in the subcultures derived from the various enrichment media showed little variation. In all subcultures with unclear colony morphologies all suspicious morphotypes were picked and tested by direct colony real-time PCR targeting a specific sequence of the *B*. *pseudomallei* TTSS1 gene [20].

### Bacterial DNA extraction and specific *B*. *pseudomallei* TTSS1 real-time PCR assay

Frozen bacterial soil culture pellets were thawed and bacterial pellets were suspended into 200μl lysis buffer (100mM Tris HCl, 100mM Na_2_EDTA, 1.5M NaCl, 1% cetyltrimethyl ammonium bromide (CTAB), pH 8.0), 10μl lysozyme (50mg/ml) and 2.5μl protease K (10mg/ml). After incubation at 37^°^C for 30 min 50μl SDS (20% w/v) was added and the tubes were further incubated at 65^°^C for 2h. An equal volume (265μl) of chloroform: isoamyl alcohol (49:1) was added into the tube. Following a mixing step (30x inversions by hand), the mixtures were centrifuged at 16,000g for 5min and 100μl of the upper aqueous phases were transferred into a new tube. To precipitate the bacterial DNA, the tubes were added with 60μl of cold isopropanol and further incubated at -20^°^C for at least 2h. The precipitated DNA was collected by centrifugation at 16,000g at 4^°^C for 5min and washed two times with pre-chilled 70% ethanol. The bacterial DNA was resuspended in 50μl of nuclease free water. The tubes were left at room temperature for 1h and DNA quality was assessed using the NanoDrop 2000 (Thermo Scientific, USA). The DNA samples were stored at -20^°^C for further real-time PCR assay.

To quantify *B*. *pseudomallei* DNA in the culture supernatants, the specific TTSS1 gene real-time PCR assay was applied [20]. A final volume of 12.5μl PCR reaction mixture consisted of 2x Maxima Probe qPCR Master Mix (Thermo Fisher Scientific, USA) and 1 μl of the bacterial DNA as template. The master mix is composed of Maxima Hot Start Taq DNA Polymerase, uracil DNA glycosylase (UDG), 400nM of the primers BpTT4176 forward and BpTT4290 reverse; 260nM of BpTT4208 probe (IDT, USA) labeled with 6-carboxyfluorescein (FAM) at its 5’ end and Black Hole Quencher 1 (BHQ1) at its 3’ position as well as 10 μg non-acetylated bovine serum albumin (BSA; AM2616, Ambion, USA). Amplification and detection were performed on the Mastercycler ep realplex real-time PCR system (Eppendorf, Germany) using the manufacturer’s standard settings. Thermal condition was set to 50°C for 2min to activate uracil DNA glycosylase (UDG), which prevents carry-over contamination, followed by an initial denaturation step at 95°C for 10 min, and 40 cycles with denaturation at 95°C for 15s and amplification at 60°C for 1min. The *B*. *pseudomallei* DNA load in the culture supernatants were expressed as cycle threshold (*C*_*T*_) values automatically calculated by noiseband algorithm and automatic baseline setting in the realplex software version 2.2. Positive real-time PCR results were defined as the appearance of the exponential fluorescence curves above the threshold. Samples with *C*_*T*_ values above 35 were repeated and only considered positive if the technical duplicate also exhibited a comparable *C*_*T*_ value.

### Statistical analysis

Statistical analyses and calculations were performed with GraphPad Prism software version 7.04 (GraphPad Software, San Diego, CA, USA). *C*_*T*_ values of TTSS1-qPCR in the text are given in brackets accompanied by the quartiles q1 and q3. Significant differences among *C*_*T*_ values of the performed TTSS1-qPCR across all applied media compositions were assessed using the Friedman´s test for non-parametric data. To perform the Friedman test despite incomplete data sets caused by the detection limit of the qPCR technique, non-detectable values were dedicated to an arbitrarily *C*_*T*_ value. *C*_*T*_ 39 was chosen since the highest measured *C*_*T*_ among all enrichments had a value of 38.81. Medians were calculated from detectable *C*_*T*_ values only. Beside the Friedman´s test, the Spearman’s rho analysis was applied to examine the correlation between *C*_*T*_ values and culture positivity. Additionally, Fisher's exact tests was used to identify significant differences between the numbers of culture or *C*_*T*_ positive samples among the various culture conditions.

## Results

### Erythritol as a single carbon source promotes growth of *B*. *pseudomallei*, but not of *B*. *thailandensis* and many other related bacteria

**Fig 1:**
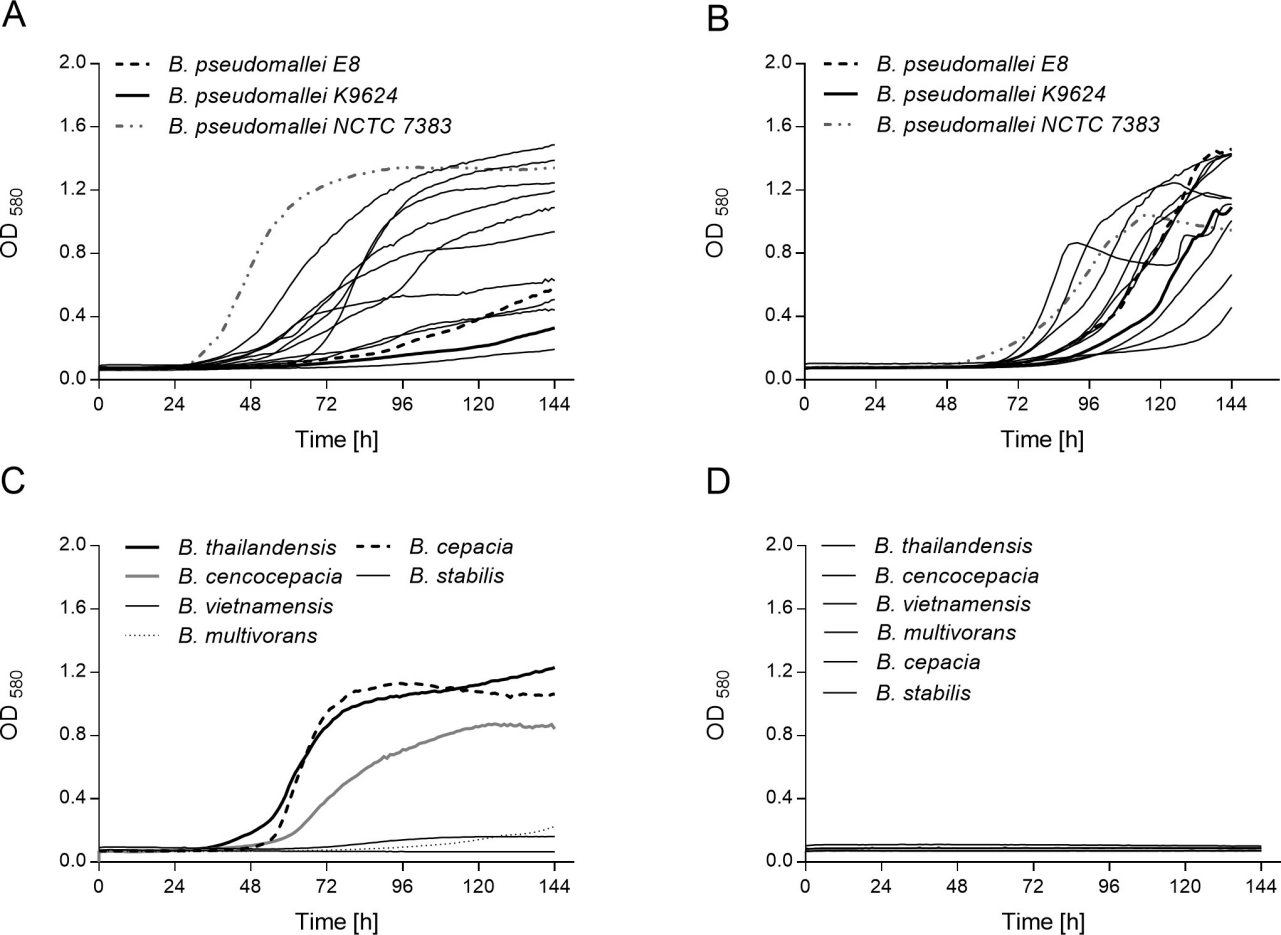
Growth of *Burkholderia pseudomallei* and other soil- dwelling bacteria in TBSS-C50 and erythritol medium (EM). Different *B*. *pseudomallei* and several other soil-dwelling bacterial strains were cultivated for 144h at 40°C in TBSS-C50, EM or EM without colistin. Bacterial growth was monitored by measuring the absorbance of the cultures at 580nm hourly. **(A)** Growth of *B*. *pseudomallei* strains in TBSS-C50. In the interest of clarity, only the isolates K9624, E8 and NCTC 7383 mentioned in the text are specifically labelled. The remaining nine tested isolates are listed in Table 1. **(B)** Growth of *B*. *pseudomallei* strains in EM. **(C)** Growth of *non- B*. *pseudomallei* strains in TBSS-C50. **(D)** Growth of *non- B*. *pseudomallei* strains in EM medium without colistin. Each growth curve is representative of two independent experiments with similar results. Each experiment was conducted in technical duplicates.

Currently recommended liquid media for the isolation of *B*. *pseudomallei* from the environment contain antibiotics such as colistin and gentamicin to enhance selectivity. However, many other soil bacteria, among them *Burkholderia* spp. share the mentioned resistance against the latter substances and can grow in TBSS-C50 and on Ashdown agar used for subcultures. This lack of specificity can lead to overgrowth of *B*. *pseudomallei* by other soil bacteria and has a significant effect on sensitivity. Previous investigations demonstrated the ability of *B*. *pseudomallei* to metabolize erythritol in contrast to related *Burkholderia* species such as *B*. *thailandensis* [9]. We therefore modified TBSS-C50 medium by adding erythritol and (NH_4_) H_2_PO_4_ as carbon and nitrogen source respectively, to replace L-threonine and nitrilotriacetic acid. This medium was designated EM. As shown in Fig 1B, after a lengthy lag phase *B*. *pseudomallei* can grow in EM with erythritol as the single carbon source; whereas all other tested soil-dwelling *Burkholderia* species are not able to utilize this sugar alcohol (Fig 1D). In contrast, a number of the non- *B*.*pseudomallei* species tested including *B*. *thailandensis* can grow in TBSS-C50 containing colistin (Fig 1C). Our experiments also indicate, that some *B*. *pseudomallei* strains like E8 and K9624 seem to have shorter lag periods in EM compared to TBSS-C50, whereas there are other *B*. *pseudomallei* strains like strain NCTC 7383 which seem to adapt faster to growth conditions in TBSS-C50 medium (Fig 1A and 1B).

### Comparison of TBSS-C50 and EM for *B*. *pseudomallei* enrichment from rice paddy soils

**Fig 2:**
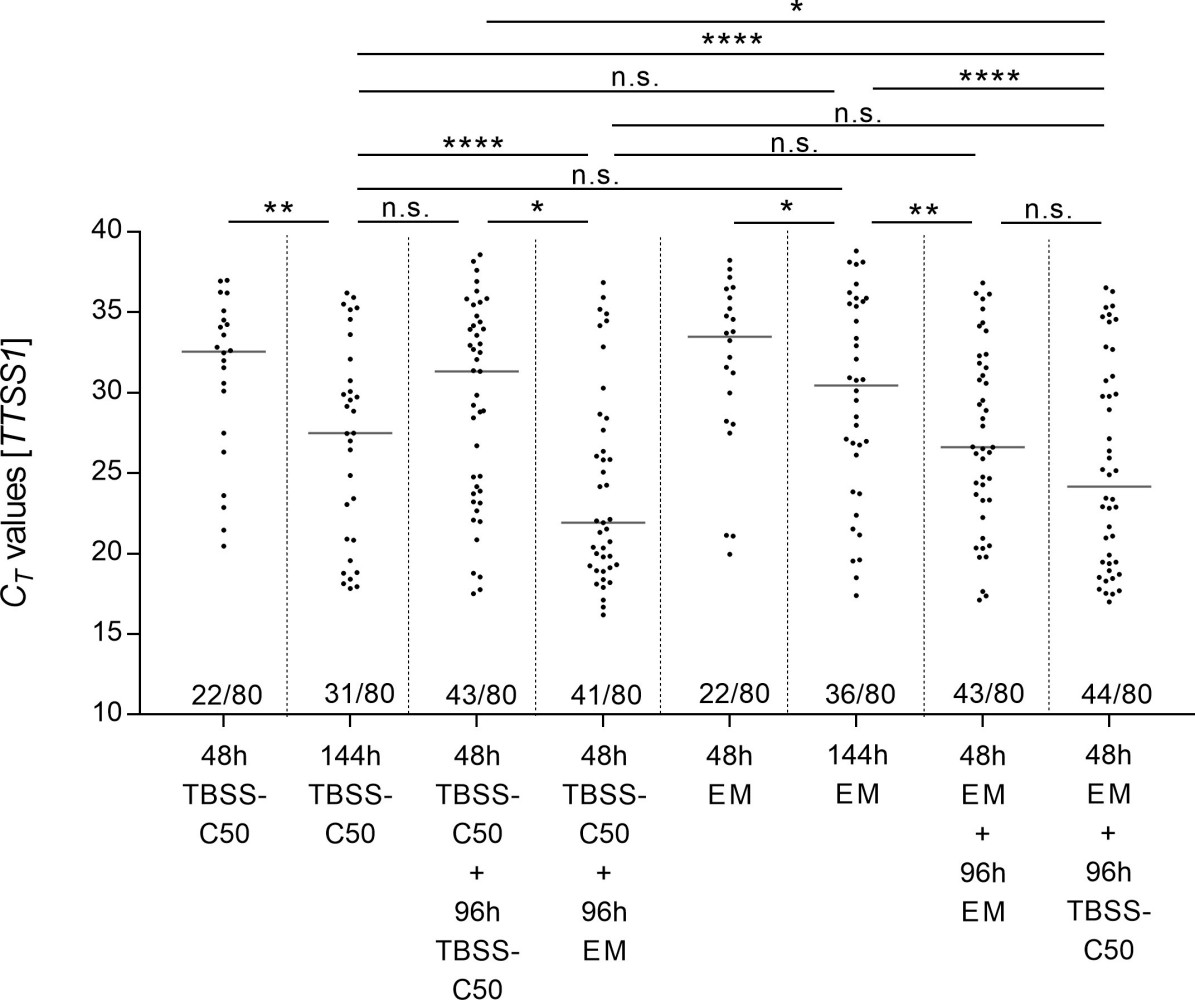
Enrichment of *B*. *pseudomallei* from soil samples using different culture conditions. Eighty soil samples collected from 16 rice paddies in different parts of Vietnam were subjected to eight different culture conditions using either TBSS-C50 or EM in single step cultures (48h or 144h) or in two step cultures with both media in various combinations (48h plus 96h) as indicated. *B*. *pseudomallei* growth was assessed by *B*. *pseudomallei*-specific qPCR at the end of each incubation of those 640 cultures. Detectable qPCR cycle threshold (*C*_*T*_) values with corresponding medians of 51 soil samples cultured in different enrichment methods are plotted in the graph. Each dot represents a single sample. The total numbers of qPCR-positive samples for each culture condition are shown below the corresponding dots above the abscissa. Significance analyses between the *C*_*T*_ values were performed using the Friedman´s test with a *C*_*T*_ value of 39 designated to qPCR-negative supernatants (*p<0.05.; **p<0.01; ***p<0.001; ****p<0,0001).

Having shown that all *B*. *pseudomallei* strains tested grow in erythritol medium EM whereas near relatives such as *B*. *thailandensis* cannot, we aimed to test the usefulness of this medium to specifically promote growth of *B*. *pseudomallei* in soil samples. In order to validate if indeed EM provides a greater enrichment for *B*. *pseudomallei*, we tested a variety of single and two-step culture approaches with 80 soil samples collected from 16 rice paddies in north and south central parts of Vietnam. Aliquots of all 80 homogenized soil samples were incubated under the same culture conditions. As shown in Fig 2, we compared single step (48h and 144h) and two step cultures (first step 48h, second step 96h) of soil samples in TBSS-C50 or in EM and with both media in combination. Culture supernatants of eight culture conditions of 80 samples (640 supernatants) were analyzed by a *B*. *pseudomallei*-specific qPCR at the end of each incubation time. 63.8% (51) soil samples gave a positive qPCR signal in at least one of the tested enrichment methods. 36.2% (29) soil samples were qPCR negative in all enrichments.

### Soil enrichments in TBSS-C50 or EM for 144h resulted in higher *B*. *pseudomallei* loads and higher number of qPCR-positive supernatants compared to TBSS-C50 for 48 h

Compared to the median *C*_*T*_ values of 48h TBSS-C50 (32.55, q_1_ = 27.2, q_3_ = 34.66) and 48h EM (33.48, q_1_ = 28.2, q_3_ = 36.05) incubation, the *C*_*T*_ values of 144h in TBSS-C50 (27.49, q_1_ = 20.85, q_3_ = 32.08; p< 0.01) and 144h in EM broth (30.46; q_1_ = 24.42, q_3_ = 35.63; p< 0.05) were significantly lower (Fig 2). In line with these observations, a higher number of qPCR-positive supernatants among the 80 enriched soil samples could be detected by qPCR after longer enrichments: 48h in either TBSS-C50 or EM resulted in 27.5% (22/80) qPCR positive samples, whereas 144h enrichment in TBSS-C50 resulted in 38.75% (31/80, n.s.) and 144h in EM resulted in 45% (36/80, p<0.05) (Fig 2). However, there was no significant difference between both media when incubated for the same period.

### Two-step enrichments including EM lead to the highest *B*. *pseudomallei* loads

As shown in Fig 2, a two-step culture either starting with TBSS-C50 for 48h followed by EM for 96h (21.93; q_1_ = 19.2, q_3_ = 28.05) or the other way round (24.18; q_1_ = 19.42, q_3_ = 30.95) was superior in terms of *B*. *pseudomallei* load shown by lower median *C*_*T*_ values compared to a 144h single step incubation of TBSS-C50 (27.49; q_1_ = 20.85, q_3_ = 32.08; for both two-step cultures p < 0.0001) and a 144h single step incubation of EM (30.46; q_1_ = 24.42, q_3_ = 35.63; for both two-step cultures p < 0.0001). There was no significant difference between both two-step combinations in the positivity rate: 51% (41/80) in TBSS-C50 48h plus EM 96h) versus 55% (44/80) in EM 48h plus TBSS-C50 96h, nor in the median *C*_*T*_ values expressing the *B*. *pseudomallei* load (TBSS-C50 48h plus EM 96h: 21.93, q_1_ = 19.2, q_3_ = 28.05; EM 48h plus TBSS-C50 96h: 24.18, q_1_ = 19.42, q_3_ = 30.95).

To control for a simple nutrient effect of the transfer of the 48h culture to a second unconsumed culture medium, we included two- step cultures in the same medium in our experimental design (Fig 2). The two-step with TBSS-C50 48h plus TBSS-C50 96h (31.33; q_1_ = 23.74, q_3_ = 34.41) led to a significantly lower enrichment of *B*. *pseudomallei* compared to the combinations of both TBSS-C50 and EM in either order (for both two-step combinations p < 0.05) (Fig 2). Though the median *C*_*T*_ values of the two-step settings with TBSS-C50 and EM in combination were lower, there was no significant difference in the molecular load compared to EM 48h plus 96h (26.62; q_1_ = 23.31, q_3_ = 31.83).

### Two step enrichment with TBSS-C50 followed by EM led to the highest recovery of *B*. *pseudomallei* isolates on Ashdown agar

**Fig 3:**
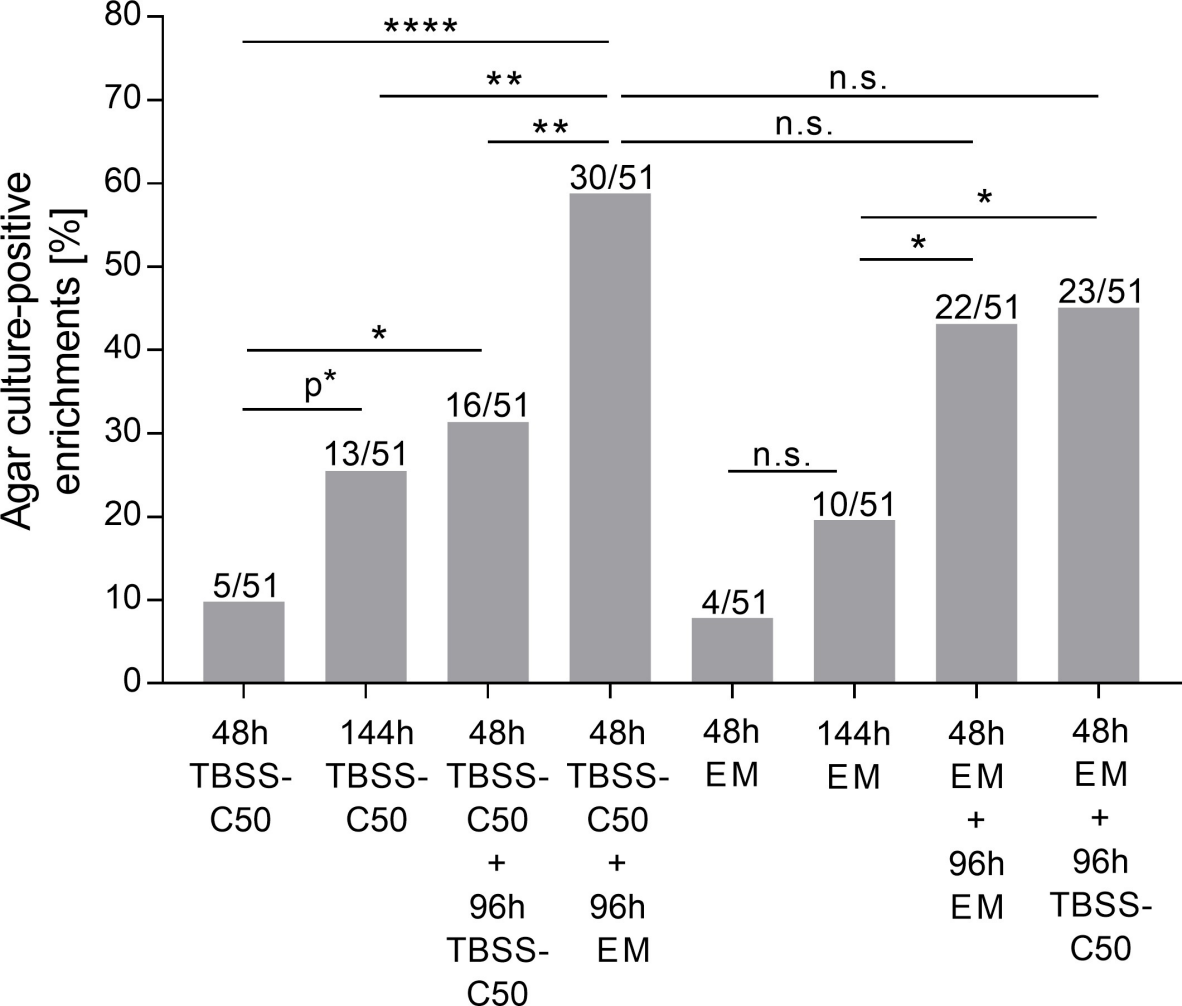
Impact of the various enrichment methods on the number of *B*. *pseudomallei* culture positive samples on Ashdown agar. All supernatants of the 51 samples with a positive qPCR signal in at least one of the enrichments (408 supernatants) were subcultured on Ashdown agar. The percentage of Ashdown agar culture-positive enrichments among all 51 samples is shown as grey bars for each culture condition. The total number of *B*. *pseudomallei* culture-positive samples in the respective culture condition is written above the corresponding bar. Statistical analyses were performed using the Fisher's exact test (*p<0.05; **p<0.01; ****p<0,0001, p* = 0.067).

To determine the impact of the enrichment protocols on the growth of *B*. *pseudomallei* on Ashdown agar from subcultures, supernatants of all 8 culture conditions of those 51 samples that were qPCR-positive in at least one enrichment method (408 supernatants) were streaked on Ashdown agar. As shown in Fig 3, significantly higher percentages of *B*. *pseudomallei* agar culture-positive samples were obtained from the two-step cultures using TBSS-C50 followed by EM (58.8%) compared to TBSS-C50 for 48h (9.8%, p<0.0001) and 144h (25.5%, p<0.01) and 48h plus 96h (31.4%, p<0.01).

**Fig 4:**
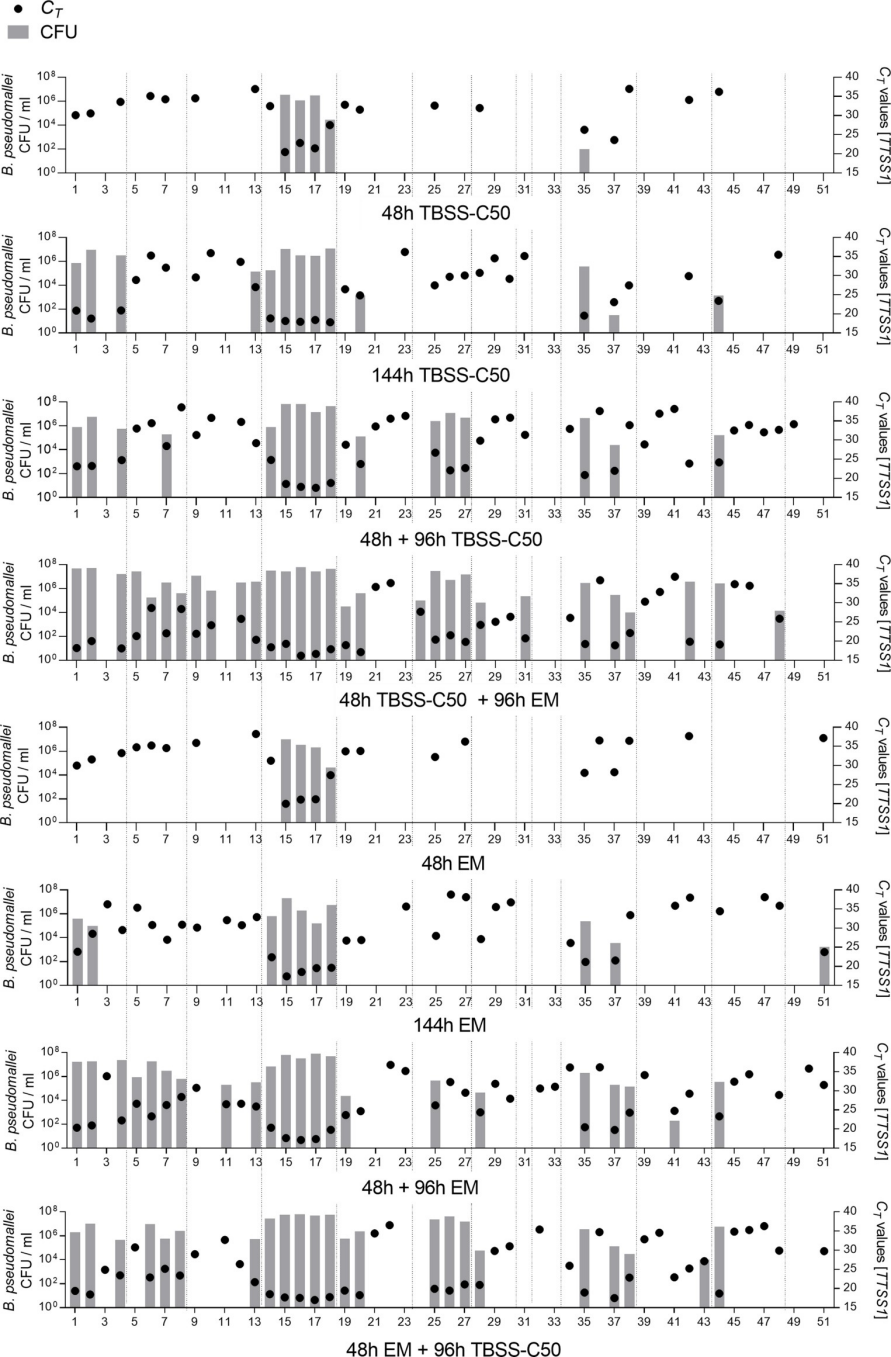
Impact of the various enrichment methods on the *B*. *pseudomallei* load and subsequent recovery on Ashdown agar in single samples. From 80 soil samples incubated under eight different culture conditions using TBSS-C50 and the erythritol containing EM broth, 51 soil samples gave a positive qPCR signal in at least one enrichment. All supernatants of the different culture conditions from these 51 soil samples were subcultured on Ashdown agar at the end of the respective incubation time. Dotted vertical lines frame the soil samples of the same rice field. A single graph combines the data of all soil samples cultured under one respective culture condition. Colony counts of *B*. *pseudomallei* are log10-transformed and plotted as bars (refer to left Y axis). Counts were calculated as geometric means of duplicates cultured from each supernatant. Corresponding cycle threshold (*C*_*T*_) values for each culture condition are visualized as dots within the bars (right Y axis).

Fig 4 depicts the association of *C*_*T*_ values determined in the various enrichment media and the *B*. *pseudomallei* counts recovered on Ashdown agar on a single sample level. Shown are the 51 samples with at least one positive signal in one of the enrichments, derived from 13 rice fields. All samples from three rice fields were qPCR negative and were not subcultured on Ashdown agar. Low *C*_*T*_ values of the enrichment cultures correlated strongly with culture positivity on Ashdown agar (r (408) = -0.85, p< 0.0001, Spearman correlation between *C*_*T*_ values and culture counts from all cultures). The *C*_*T*_ values of the 123 culture supernatants from which *B*. *pseudomallei* isolates were recovered on agar ranged between *C*_*T*_ 16.2 and *C*_*T*_ 28.67 (median *C*_*T*_ 20.97; q_1_ = 18.83, q_3_ = 23.74).

Considering all enrichment conditions, in 34 out of the 51 (66,66%) samples, subcultures grew *B*. *pseudomallei* on Ashdown agar (Fig 4). 17 (50%) soil samples that were *B*. *pseudomalle*i agar culture-positive, were only detected from enrichment protocols including EM and would have been missed by only TBSS-C50 use. Seven of those 17 samples where only positive in the two-step protocol TBSS-C50 48h plus EM 96h, one sample was only positive in the reversed order of both media, another two in the EM 48h plus 96h two step and one sample after incubation in EM for 144h (Fig 4). There was no sample being only agar culture-positive when enriched in TBSS-C50 medium, but negative from enrichment in EM.

## Discussion

In many tropical and subtropical countries, which have been predicted to be endemic for melioidosis, the environmental detection of its causative agent *B*. *pseudomallei* is still pending.

A number of factors determining the presence of this pathogen such as high rainfall and soil water content, temperature [1, 21], soil modified by human activities [1, 22], clay content in soil [1, 23, 24], salinity [1, 22, 23, 25, 26] and the proportion of gravel [1, 27], have been associated with the presence of *B*. *pseudomallei*. Most likely, several factors determining the variable abundance of *B*. *pseudomallei* in a small geographical scale still need to be identified. In this context cultural detection of *B*. *pseudomallei* in the environment will remain an important method to define melioidosis risk areas, especially in low-income countries because of considerably lower expenses compared to molecular methods.

The cultural identification of certain single bacterial species from a soil habitat with approximately 10^9^ different organisms per gram is very challenging and faces multiple methodological difficulties including the possibility of viable but non-culturable organisms [28–30]. Consequently, direct molecular methods have been widely applied in many studies [31] including the characterization of the soil microbiome in rice paddy soils [32–34]. The current consensus guidelines for the isolation of *B*. *pseudomallei* from environmental samples [4] were an important first attempt to standardize the culture attempts, although proposed parameters such as incubation times, optimal media composition etc. have not been validated in respective studies. The fact that the current protocols also enrich non-*B*. *pseudomallei* bacteria to a high extent is a particular problem with *B*. *thailandensis*. *B*. *thailandensis* shares many phenotypic characteristics, including a similar colony appearance on Ashdown agar [35], the particular resistance pattern [36] but not the virulence [37, 38]. Both species may co-exist in the environment [39] leading to false-negative results for *B*. *pseudomallei* due to the potential overgrowth of *B*. *thailandensis*. Previous studies from Thailand and Vietnam have shown that the majority of *B*. *pseudomallei* culture-negative soils from Thailand and Vietnam grew *B*. *thailandensis* [39–42].

This study therefore aimed to develop and validate a more selective soil culture protocol by targeting erythritol catabolism of *B*. *pseudomallei*. A relatively small number of microorganisms is known to have the capability to assimilate erythritol [9]. Among those are *Brucella* spp. in which the responsible metabolic gene clusters have been characterized [43, 44]. Our growth experiments with *B*. *pseudomallei* strains and other related bacteria revealed that our novel erythritol medium supports the growth of *B*. *pseudomallei* and, most importantly, the medium does not support growth of related bacteria, among them *B*. *thailandensis* (Fig 1). Though EM supported growth of all tested *B*.*pseudomallei* strains, relatively long lag phases of > 48 hours were noticeable. Although mutational events cannot be excluded, the extended lag periods were most likely caused by transcriptional adaptations of the metabolic pathways in the small culture volumes used in the microtitre plates in the presence of colistin (Fig 1). Own studies have recently identified the genes and metabolites involved in the catabolism of erythritol by *B*. *pseudomallei* (to be published elsewhere).

The evaluation of different incubation times and combinations of our new EM and the standard medium TBSS-C50 with rice paddy soils revealed the following: Firstly, incubation of soil cultures for 144h in either TBSS-C50 or EM resulted in increased *B*. *pseudomallei* growth and qPCR-positive samples compared to the currently recommended TBSS-C50 for 48h (Fig 2). Secondly, a two-step enrichment approach with 48h of TBSS-C50 medium followed by 96h of erythritol medium EM showed the greatest growth and resulted in a six times higher yield of *B*. *pseudomallei-*positive samples on Ashdown agar in comparison to the consensus guideline culture TBSS-C50 for 48h (Fig 3). The positive effect of the two-step enrichment might be due to the dilution of carbon sources derived from soil in the first culture, increasing the selectivity of the second EM culture step.

The single medium two-step enrichment TBSS-C50 48h plus TBSS-C50 96h led to only approximately half the number of *B*. *pseudomallei* Ashdown agar-positive samples obtained with the TBSS-C50 48h plus EM 96h enrichment. Our results indicate that the higher selectivity of EM has the potential to significantly reduce the high rate of false-negative culture results associated with the current standard protocols. However, even with this improved protocol approximately 40% of PCR-positive samples remained culture-negative, indicating still a significant gap between both methods. In a previous study we used polyethylene glycol and sodium deoxycholate to detach *B*. *pseudomallei* cells from soil particles to allow a more sensitive direct cultural quantification on Ashdown medium without enrichment [45]. In this study we did not combine the detachment protocol with our new enrichment protocol, since we wanted to exclude that polyethylene glycol might act as an alternative carbon source and hence reduces the selective effect of Erythritol. However, if a combination of the detachment protocol and our new enrichment protocol can further improve cultural detection, is worth to be tested in future studies.

Since a number of microorganisms, including for instance plant-associated lactic acid bacteria [46], have been shown to produce erythritol and this sugar alcohol appears to be a storage substance in fungi [47–50], lichens [51] and algae [52, 53] it remains to be determined if *B*. *pseudomallei* might co-exist with such organisms and if the ability to metabolize erythritol might be a selective advantage in a competitive and highly diverse environment. Future studies also have to demonstrate if the higher sensitivity of our two-step TBSS-C50/EM culture approach holds true for non-rice paddy soils and other environmental samples such as river water with very different physicochemical properties and microbial compositions.
